# Ebola could be eradicated through voluntary vaccination

**DOI:** 10.1098/rsos.171591

**Published:** 2018-01-24

**Authors:** Andrew Brettin, Rosa Rossi–Goldthorpe, Kyle Weishaar, Igor V. Erovenko

**Affiliations:** 1 Department of Mathematics, University of Minnesota—Twin Cities, Minneapolis, MN 55455, USA; 2 Department of Mathematics, Bowdoin College, Brunswick, ME 04011, USA; 3 Department of Mathematics, Regis University, Denver, CO 80221, USA; 4 Department of Mathematics and Statistics, University of North Carolina at Greensboro, Greensboro, NC 27402, USA

**Keywords:** Ebola, epidemiology, game theory, Nash equilibrium, uncertainty and sensitivity analysis, vaccination

## Abstract

Ebola virus disease (EVD) is a severe infection with an extremely high fatality rate spread through direct contact with body fluids. A promising Ebola vaccine (rVSV-ZEBOV) may soon become universally available. We constructed a game-theoretic model of Ebola incorporating individual decisions to vaccinate. We found that if a population adopts selfishly optimal vaccination strategies, then the population vaccination coverage falls negligibly short of the herd immunity level. We concluded that eradication of Ebola is feasible if voluntary vaccination programmes are coupled with focused public education efforts. We conducted uncertainty and sensitivity analysis to demonstrate that our findings do not depend on the choice of the epidemiological model parameters.

## 1. Introduction

Ebola virus disease (EVD), formerly known as Ebola haemorrhagic fever, is an acute and often fatal viral infection caused by viruses of the genera *Ebolavirus* from the family *Filoviridae* [1]. It is transmitted through direct contact with body fluids (blood, urine, saliva, sweat, faeces, vomit, breast milk and semen) from infected or recently deceased infectious individuals, contaminated objects (needles and syringes) and wild animals (fruit bats and primates) [1,2]. Those who contract the disease experience flu-like symptoms for the first 1–3 days of the infection, such as fever, headache, muscle aches and fatigue [3]; which are later followed by additional afflictions, like vomiting, diarrhoea, abdominal pains, loss of appetite and haemorrhages [1,3,4]. The disease has a high case-fatality ratio and significant potential to develop into an epidemic [5]. The World Health Organization estimates the fatality rate of those infected to be approximately 50% [1], with rates for past outbreaks ranging from 25% to 90% [5,6].

After initial exposure, there is a 2–21-day latency period before symptoms develop [4,7]. It is estimated that this incubation period lasts 8–10 days, on average [4]. As soon as symptoms emerge, individuals are capable of transmitting the disease [2].

The 2014–2016 outbreak in West Africa was the largest Ebola outbreak ever recorded [1,3,5,8,9]. It is believed that the first case occurred in Guinea during December of 2013 [10]. The disease infected over 28 000 people and caused at least 11 300 deaths [11], exceeding the number of cases and mortalities from all other Ebola outbreaks combined [1,5]. In addition to the personal losses dealt to families and communities, the disease ravaged the overall health and economic systems of the three most affected countries (Guinea, Liberia and Sierra Leone) [3]. The epidemic received widespread press and engendered heavy international support for victims of the disease.

One noteworthy aspect of Ebola outbreaks is that burial rituals, which involve direct contact with the deceased, tend to serve as a common medium of transmission [1,8,12]. For instance, in the 1995 epidemic in the Democratic Republic of Congo, exposure to infected corpses caused two-thirds of new infections [13]. During the West Africa epidemic, 60% of EVD cases in Guinea were linked to traditional funeral rites [14,15]. In Sierra Leone, local health authorities traced as many as 365 deaths to participation in the funeral of one traditional healer [15].

One principal reason the West Africa epidemic was so large is that rigid cultural norms and traditional belief systems, which tend to make individuals less likely to adopt safe practices, allowed for rapid proliferation of the disease. Many people in West Africa simply deny the existence of Ebola, believing it to be government propaganda made to collect foreign aid dollars or manipulate the population [16,17]. To some, Ebola is seen more as a ‘curse’ rather than a virus; as a result, family members are reluctant to help or encourage loved ones to receive treatment [16], increasing their risk of exposure to the disease. Others refuse to be quarantined out of fear that the government might deliberately infect them during quarantine [17]. This mistrust for government, rooted in a history of corruption and political instability [18], even prompted some citizens to stone and kill aid workers [19], further impeding measures to contain EVD. Additionally, the inclination to give deceased family members traditional burials has prompted many to host burials in secret [16,17] and abstain from contacting special burial teams trained to properly and safely dispose of contaminated bodies [16]. Evidently, strong traditional belief systems and customs generally increase transmission rates and accelerate spread of the disease in Ebola epidemics.

The West Africa outbreak impelled clinical development of an Ebola vaccination [20]. As of now, the recombinant Vesicular Stomatitis Virus Zaire Ebolavirus vaccine (rVSV-ZEBOV) is the only vaccine that passes safety and immunogenicity tests [21]. In a recent ring vaccination clinical trial, rVSV-ZEBOV had an estimated efficacy rate between 74.7% and 100% [22]. Although it is still unknown how long the vaccine protects susceptible individuals and how effective the vaccine will be when distributed on a large scale, no cases of EVD have occurred 10 or more days following vaccination of individuals in clinical trials.

A significant number of mathematical models have been developed to describe the transmission dynamics of EVD [3,13,17,23–25]. Ebola models are typically based on SEIR epidemiological models, in which susceptible, exposed (but not infectious), infected (and infectious) and recovered (SEIR) individuals are divided into homogeneous compartments. EVD transmission owing to contact with infectious corpses has been accounted for in different ways. Some models incorporate a separate compartment for infectious corpses [3,13,17,24,25], while others boost the transmission rate owing to infectious living individuals or increase the duration of infection [23,26,27].

To the best of our knowledge, no existing epidemiological models of Ebola incorporate vaccination, mainly owing to the lack of an approved vaccine. Here, we constructed such a model by adopting a version of the model in [3] and adding a compartment for vaccinated individuals. Such epidemiological models can be used to determine mandatory vaccination programmes. However, for many infectious diseases, preventive actions rely on voluntary individual participation rather than mandatory policies. Individual vaccination decisions are often informed on what the rest of the population does; such strategic decisions can be framed within game-theoretic models.

Game theory has long been used to model various biological phenomena [28–30]. In particular, a game-theoretic framework has been applied to vaccination decisions [31]. It accounts for the probability of infection of a focal individual given the vaccination decisions of the rest of the population, as well as for the (perceived) cost of vaccination versus the cost of the infection. This approach has been used to study individual vaccination decisions for smallpox [32], influenza [33], measles [34], rubella [35] and toxoplasmosis [36]. This modelling framework can also be adapted to address other types of interventions such as insecticide-treated cattle to eliminate African sleeping sickness [37], mosquito repellent to combat dengue fever [38] and insecticide-treated bed nets to fight malaria [39]. See also [40] for a review of recent applications of statistical physics methods to vaccination decisions.

In this paper, we construct a game-theoretic model of individual decisions to vaccinate for Ebola. We first construct an epidemiological compartment model of Ebola with a compartment for vaccinated individuals. We use this model to compute the disease-free equilibrium, the endemic equilibrium, the basic reproduction number and the herd immunity threshold vaccination rate. We then let individuals choose whether to vaccinate or not vaccinate. Individuals weigh the probability of getting infected, which depends on the vaccination decisions of the rest of the population, and the cost of vaccination versus the cost of infection. We compute the Nash equilibrium vaccination rate of the population by comparing the pay-offs of the two strategies (to vaccinate or to not vaccinate). The fundamental question we are interested in is this: if the population adopts the optimal (Nash equilibrium) vaccination rate, would it be possible to eradicate the disease? We find that the optimal vaccination rate falls short of the herd immunity vaccination rate, which is a typical outcome for similar models of many other diseases. However, what makes our Ebola model unique is the high fatality rate of the disease (i.e. an extremely high cost of infection). This forces the optimal vaccination rate to be very close to the herd immunity vaccination rate, and we infer that adding additional measures, such as public education initiatives, may be sufficient to push the voluntary vaccination rate to the herd immunity level.

## 2. Material and methods

**Figure 1.**
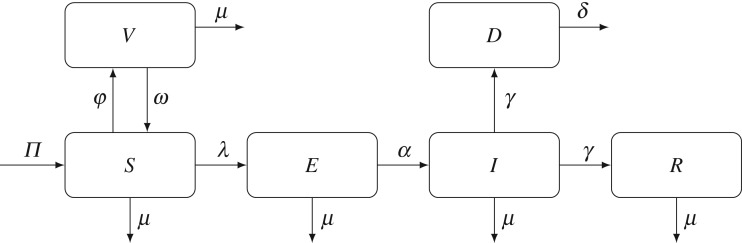
Compartmental model of Ebola with vaccination. The population is divided into five compartments: susceptible (*S*), vaccinated (*V*), exposed (*E*), infected (*I*) and recovered (*R*) individuals. Corpses of recently deceased but still infectious individuals correspond to the *D* compartment. Susceptible individuals vaccinate at the rate *φ* and acquire temporary immunity from the disease; immunity wanes at the rate *ω*.

We adopted a slightly simplified version of the epidemiological model of Ebola from Agusto [3] and included a compartment for vaccinated individuals; see figure 1 for the model diagram. The entire population (*N*) is divided into five homogeneous compartments—susceptible (*S*), vaccinated (*V*), exposed (*E*), infected (*I*) and recovered (*R*) individuals. We also account for corpses of recently deceased but still infectious individuals in the *D* compartment.

Individuals enter the population at a constant rate *Π*, by birth or immigration, and leave the population at a constant rate *μ* representing death owing to natural causes. Individuals in the susceptible class enter the vaccinated class at a rate *φ*. Preliminary studies have shown the rVSV-ZEBOV vaccine to be highly effective [41], and no individuals in clinical trials have contracted the disease 10 days or more after the vaccination. Therefore, we assumed that the vaccine is 100% effective: an individual residing in the *V* compartment cannot contract Ebola. To compensate for this possibly over-optimistic assumption, we considered the worst case scenario for the duration of the immunity granted by the vaccine. The effect of the vaccine wears off at a rate *ω*, and our baseline value of this parameter is chosen so that the immunity wanes on average in one year.

Susceptible individuals contract Ebola at a rate λ. Following [3,17], we have

2.1 $$\lambda =\frac{\beta \xi (I+\tau D)}{N},$$

where *β* is the base effective contact rate, *τ* is a modification parameter accounting for differences in infectiousness between infected and deceased individuals, and *ξ* is a modification parameter which accounts for the strength of cultural norms in the population.

After contracting the disease, exposed individuals experience a latency period as the virus incubates. During this time, individuals are not infectious. Exposed individuals move to the infected class at a rate *α*. As individuals are approximately equally likely to die from Ebola as they are to recover [1,3], both the disease-induced death rate and the recovery rate are represented by the same parameter *γ*. Deceased individuals are no longer infectious once they are buried, which happens at a rate *δ*. Recovered individuals are known to develop antibodies lasting at least a decade [15]. While reinfection with Ebola virus is rare, it may persist in body fluids resulting in potential reactivation of the illness [42]. In our model, we assume that recovered individuals cannot be reinfected with Ebola. Table 1 contains a summary of the notation for the model parameters, and table 2 contains baseline values of the parameters used in our computations. We assume an average individual lifespan of 60 years.

**Table 1.** Summary of the parameters of the model.

symbol	description
*Π*	recruitment rate
*μ*	natural death rate
*φ*	vaccination rate
*ω*	vaccine wear-off rate
*α*	incubation rate
*γ*	recovery or death rate of symptomatic individuals
*δ*	burial rate of Ebola-deceased individuals
*β*	effective contact rate
*τ*	modification parameter for infectiousness of contaminated corpses
*ξ*	strength of traditional belief systems and customs

**Table 2.** Parameter values and ranges.

symbol	value	range	source
*Π*	400 day^−1^	(360,440) day^−1^	[17]
*μ*	(365⋅60)^−1^ day^−1^	((66⋅365)^−1^,(54⋅365)^−1^) day^−1^	assumed
*ω*	365^−1^ day^−1^	((10⋅365)^−1^,(0.5⋅365)^−1^) day^−1^	assumed
*α*	0.1 day^−1^	(1/11,1/8) day^−1^	[4]
*γ*	0.2683 day^−1^	(0.2415,0.2951) day^−1^	[17]
*δ*	0.5 day^−1^	(1/3,1) day^−1^	[17]
*β*	0.3045 day^−1^	(0.2741,0.3350) day^−1^	[17]
*τ*	0.21	(0.1,0.5)	[17,25]
*ξ*	varies	(2,5)	
*φ*	varies	(0,0.002)	

The following system of differential equations describes our epidemiological model:

2.2 $$\begin{array}{rl} \frac{dS}{dt} & =\Pi +\omega V-(\varphi +\mu )S-\lambda S,\\ \frac{dV}{dt} & =\varphi S-(\omega +\mu )V,\\ \frac{dE}{dt} & =\lambda S-(\alpha +\mu )E,\\ \frac{dI}{dt} & =\alpha E-(2\gamma +\mu )I,\\ \frac{dR}{dt} & =\gamma I-\mu R\\ \;\text{and}\;\frac{dD}{dt} & =\gamma I-\delta D. \end{array}\}$$

The disease free equilibrium (DFE) is then given by

2.3 $$(S^{0},V^{0},E^{0},I^{0},R^{0},D^{0})=\left(\frac{\Pi (\omega +\mu )}{\mu (\varphi +\omega +\mu )},\frac{\Pi \varphi }{\mu (\varphi +\omega +\mu )},0,0,0,0\right).$$

The basic reproduction number $R_{0}$ represents the expected number of secondary cases that an infected individual will cause in a purely susceptible population. Using the next-generation matrix method [43] to compute the basic reproduction number for our model of Ebola, we consider the dynamics of the classes *E*, *I* and *D*, which contribute to new infections. Let *F* be the sensitivity matrix of the appearance rate of new infections, and *V* be the sensitivity matrix of the transition rate of existing infections. Then

2.4 $$F=\left[\begin{array}{ccc} 0 & \frac{\beta \xi (\omega +\mu )}{\varphi +\omega +\mu } & \frac{\beta \xi \tau (\omega +\mu )}{\varphi +\omega +\mu }\\ 0 & 0 & 0\\ 0 & 0 & 0 \end{array}\right]\;\text{and}\;V=\left[\begin{array}{ccc} \alpha +\mu & 0 & 0\\ -\alpha & 2\gamma +\mu & 0\\ 0 & -\gamma & \delta \end{array}\right].$$

The basic reproduction number $R_{0}$ is equal to the spectral radius of the matrix *FV* ^−1^:

2.5 $$R_{0}=\frac{\alpha \beta \xi (\omega +\mu )(\delta +\gamma \tau )}{\delta (\varphi +\omega +\mu )(\alpha +\mu )(2\gamma +\mu )}.$$

As long as $R_{0}>1$, the system converges to the endemic equilibrium given by

2.6 $$\begin{array}{rl} S^{*} & =\frac{\Pi (\omega +\mu )[\alpha (\gamma +\mu )+\mu (2\gamma +\mu )]}{\mu (\varphi +\omega +\mu )[R_{0}(\alpha +\mu )(2\gamma +\mu )-\alpha \gamma ]},\\ V^{*} & =\frac{\varphi }{\omega +\mu }S^{*},\\ E^{*} & =\frac{2\gamma +\mu }{\alpha }I^{*},\\ I^{*} & =\frac{\Pi \alpha (R_{0}-1)}{\alpha (R_{0}-1)+R_{0}\mu (2\gamma +\mu )},\\ R^{*} & =\frac{\gamma }{\mu }I^{*},\\ D^{*} & =\frac{\gamma }{\delta }I^{*}\\ \;\text{and}\;N^{*} & =S^{*}+V^{*}+E^{*}+I^{*}+R^{*}=\frac{\beta \xi \alpha (\delta +\gamma \tau )}{\delta (\alpha +\mu )(2\gamma +\mu )}S^{*}. \end{array}\}$$

Note that if $R_{0}=1$, then the endemic equilibrium values are equal to the disease-free values in (2.3). Also, the endemic equilibrium expressions make biological sense only if $R_{0}>1$.

By setting $R_{0}=1$ in (2.5), we can determine the threshold vaccination rate necessary to reach herd immunity:

2.7 $$\varphi_{\mathrm{HI}}=\frac{\left(\omega +\mu )[\alpha \beta \xi (\delta +\gamma \tau )-\delta (\alpha +\mu )(2\gamma +\mu )\right]}{\delta (\alpha +\mu )(2\gamma +\mu )}.$$

**Figure 2.**
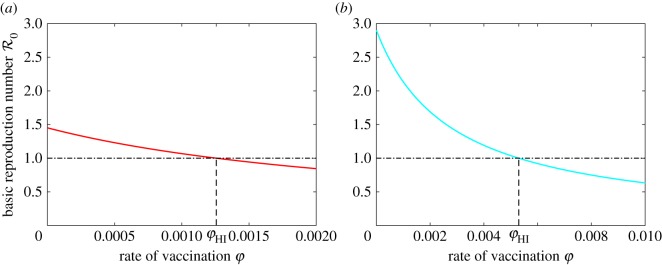
The basic reproduction number as a function of the vaccination rate *φ*. The threshold value of the vaccination rate necessary to reach herd immunity is denoted by *φ*_HI_. (*a*) *ξ*=2, which represents a community with partial adherence to traditions and customs during an epidemic; (*b*) *ξ*=4, which represents a community with stricter adherence to traditions and customs.

The graphs of $R_{0}$ as a function of the vaccination rate *φ* for two different values of *ξ* are shown in figure 2. If *φ*>*φ*_HI_, then $R_{0}<1$ and the disease is eradicated. If *φ*<*φ*_HI_, then $R_{0}>1$ and the disease remains endemic.

## 3. Results

### 3.1. Optimal vaccination strategies

In this section, we set up and solve a game-theoretic model of optimal individual vaccination strategies based on the epidemiological model of Ebola presented in figure 1. An individual has two strategies to chose from: to vaccinate or to not vaccinate. To each of the two strategies we assign an expected pay-off following the general framework of [31]:

3.1 $$\begin{array}{rl} E_{v} & =-C_{v}-\pi_{v}C_{i}\\ \;\text{and}\;E_{\mathrm{nv}} & =-\pi_{\mathrm{nv}}C_{i}, \end{array}\}$$

where *E*_v_ and *E*_nv_ are the pay-offs of individuals who chose to vaccinate or not vaccinate, respectively; *π*_v_ and *π*_nv_ are the probabilities of getting infected for a vaccinated and non-vaccinated individual, respectively; *C*_i_ is the cost of infection, and *C*_v_ is the cost of vaccination. The cost parameters include direct costs, such as the cost of the vaccine or the cost of medical treatment in case of infection; indirect costs, such as potential side effects of the vaccine or morbidity risks of the infection; and perceived costs, such as vaccine scares or underestimating the dangers of the disease.

As scaling the pay-off functions by a constant does not affect the outcome of the game, we divide both equations in (3.1) by *C*_i_ to obtain

3.2 $$\begin{array}{rl} E_{v} & =-C-\pi_{v}\\ \;\text{and}\;E_{\mathrm{nv}} & =-\pi_{\mathrm{nv}}, \end{array}\}$$

where *C*=*C*_v_/*C*_i_ is the cost of vaccination relative to the cost of infection.

To determine the probabilities of getting infected for vaccinated and non-vaccinated individuals, we use the diagram in figure 1. A non-vaccinated individual resides in the *S* compartment. It can leave the susceptible class by contracting Ebola (at a rate λ) or by dying from natural causes (at a rate *μ*). Hence the probability a non-vaccinating individual moves from the *S* compartment to the *E* compartment is λ/(λ+*μ*). Similarly, an individual residing in the *E* compartment moves to the *I* compartment with probability *α*/(*α*+*μ*). It follows that

3.3 $$\pi_{\mathrm{nv}}=\frac{\lambda }{\lambda +\mu }\;\frac{\alpha }{\alpha +\mu }.$$

A vaccinated individual may still contract Ebola if the vaccine wears off, leaving the individual susceptible. The probability of this event is *ω*/(*ω*+*μ*), and hence the probability of a singularly vaccinated individual becoming infected is

3.4 $$\pi_{v}=\frac{\omega }{\omega +\mu }\;\frac{\lambda }{\lambda +\mu }\;\frac{\alpha }{\alpha +\mu }.$$

We note that these probabilities are short-term calculations corresponding to a singular individual vaccination decision in our strategic game rather than the lifetime probabilities of infection.

We have set up the pay-off functions for the two strategies. We are now looking for conditions when an individual should vaccinate and for the optimal (Nash equilibrium) population vaccination strategy. Let *φ*_pop_ be the population vaccination rate. If *φ*_pop_>*φ*_HI_, then $R_{0}<1$, and the population reaches the disease-free equilibrium. In this case, λ=0 and the probability of getting infected is zero for any individual. It follows that a focal individual should not vaccinate, as vaccinating incurs some cost but provides no benefit: *E*_v_=−*C*<0=*E*_nv_.

If *φ*_pop_<*φ*_HI_, then $R_{0}>1$ and the population reaches the endemic equilibrium. In this case

3.5 $$\lambda =\frac{\beta \xi (I^{*}+\tau D^{*})}{N^{*}},$$

where I*, D* and N* depend on the population vaccination rate *φ*_pop_; they are given by equations in (2.6). To determine the best strategy for a focal individual when the disease is endemic, we consider the difference in pay-offs of the two strategies:

3.6 $$\Delta E=E_{v}-E_{\mathrm{nv}}=-C+\frac{\mu }{\omega +\mu }\;\frac{\lambda }{\lambda +\mu }\frac{\alpha }{\alpha +\mu }.$$

An individual should vaccinate if Δ*E*>0 and not vaccinate if Δ*E*<0. In other words, the best strategy of the focal individual depends on the prevalence of the disease (which, in turn, depends on the population vaccination rate *φ*_pop_) and the cost of vaccination relative to the cost of infection. If

3.7 $$C<\frac{\mu }{\omega +\mu }\frac{\lambda }{\lambda +\mu }\;\frac{\alpha }{\alpha +\mu },$$

then the risk of infection is higher than the relative cost of vaccination, and the individual should vaccinate. If, on the other hand

3.8 $$C>\frac{\mu }{\omega +\mu }\frac{\lambda }{\lambda +\mu }\;\frac{\alpha }{\alpha +\mu },$$

then the relative cost of vaccination outweighs the risk of infection, and the individual should not vaccinate.

The Nash equilibrium strategy is the population vaccination rate *φ*_NE_ such that no individual can improve its pay-off by deviating from this strategy. If the population is in the disease-free equilibrium, then clearly *φ*_NE_=0. If the disease is endemic, then the Nash equilibrium vaccination rate is the solution to the equation *E*_v_=*E*_nv_, that is

3.9 $$C=\frac{\mu }{\omega +\mu }\;\frac{\lambda }{\lambda +\mu }\;\frac{\alpha }{\alpha +\mu },$$

where λ is a function of the population vaccination rate; it is given by equation (3.5). Substituting the expressions for I*, D* and N* from (2.6) into (3.5) and solving the equation (3.9) for *φ*, we obtain

3.10 $$\varphi_{\mathrm{NE}}=x\;\left[\frac{\alpha \beta \xi (\delta +\gamma \tau )y-C\delta x(\alpha +\mu )z}{\delta (\alpha +\mu )(2\gamma +\mu )y}-1\right],$$

where

3.11 $$\begin{array}{r} x=\omega +\mu , \end{array}$$

3.12 $$\begin{array}{r} y=\alpha \mu -Cx(\alpha +\mu ) \end{array}$$

3.13 $$\begin{array}{r} \;\text{and}\;z=\alpha (\gamma +\mu )+\mu (2\gamma +\mu ). \end{array}$$

**Figure 3.**
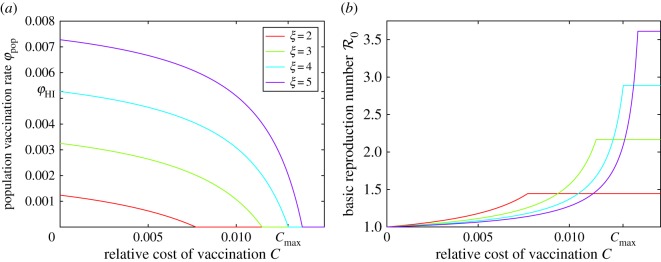
(*a*) Optimal vaccination rate *φ*_NE_ as a function of the relative cost of vaccination *C* for four different values of *ξ*. In the parameter area below each graph, an individual should vaccinate, and in the parameter area above each graph, an individual should not vaccinate. The optimal vaccination rate reaches the herd immunity threshold level *φ*_HI_ only when *C*=0: *φ*_NE_(0)=*φ*_HI_ (shown for the *ξ*=4 graph) and *φ*_NE_(*C*)<*φ*_HI_ if *C*>0. Nobody should vaccinate when the cost of vaccination relative to the cost of the infection is greater than a threshold value *C*_max_ regardless of the disease prevalence (shown for the *ξ*=4 graph). (*b*) Basic reproduction number $R_{0}$ as a function of the relative vaccination cost *C*, assuming that the population adopts the optimal vaccination rate *φ*_NE_. The value of $R_{0}$ becomes constant once the relative cost of vaccination reaches *C*_max_ (shown for the *ξ*=4 graph) because the optimal vaccination rate drops to 0 in this case.

The graphs of the optimal vaccination rate *φ*_NE_ as a function of the relative cost of vaccination *C* for four different values of the parameter *ξ* are shown in figure 3*a*. Note that the optimal vaccination rate *φ*_NE_ never exceeds the herd immunity threshold vaccination rate *φ*_HI_, and they are the same only when the cost of vaccination is zero. However, at low relative vaccination costs, the strategically optimal vaccination rate *φ*_NE_ remains close to the threshold for herd immunity. Furthermore, there is a threshold relative cost of vaccination *C*_max_ after which no individual will vaccinate.

The graphs of the basic reproduction number $R_{0}$ as a function of the relative cost of vaccination *C*, assuming the population adopts the optimal vaccination strategy, are shown in figure 3*b*. As the Nash equilibrium vaccination rate remains close to the herd immunity threshold vaccination rate for small relative vaccination costs, the basic reproduction number $R_{0}$ remains very close to 1 as long as the relative vaccination cost is sufficiently small.

### 3.2. Uncertainty and sensitivity analysis

We performed the uncertainty and sensitivity analysis [44,45] of both epidemiological and game-theoretic models. Table 2 presents the ranges and baseline values of the model parameters. We considered three response functions for the uncertainty and sensitivity analysis: (i) the basic reproduction number $R_{0}$ from the epidemiological model; (ii) the optimal vaccination rate *φ*_NE_ from the game-theoretic model and (iii) the relative difference between the herd immunity vaccination rate and the Nash equilibrium vaccination rate (*φ*_HI_−*φ*_NE_)/*φ*_HI_.

**Figure 4.**
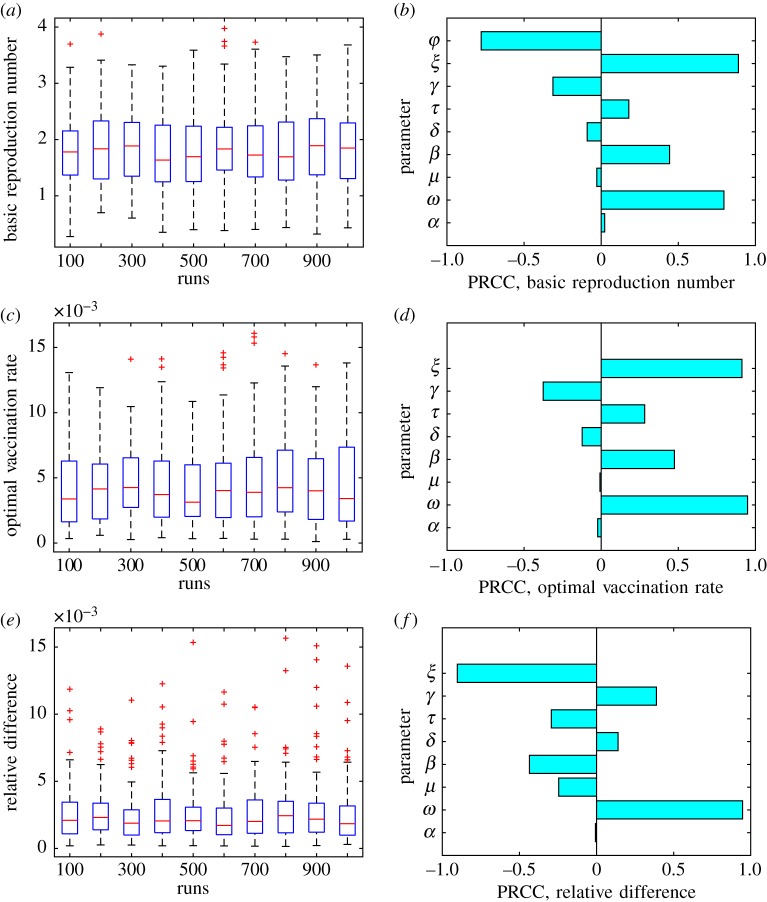
Results of the uncertainty (*a*,*c*,*e*) and sensitivity (*b*,*d*,*f*) analysis. Three response functions were considered: (i) the basic reproduction number $R_{0}$ (*a*) and (*b*); (ii) the optimal vaccination rate *φ*_NE_ (*c*) and (*d*) and (iii) the relative difference between the herd immunity vaccination rate and the optimal vaccination rate (*φ*_HI_−*φ*_NE_)/*φ*_HI_ (*e*) and (*f*). The value *C*=10^−4^ was used for the response functions (ii) and (iii).

We sampled 1000 values of each model parameter from the intervals listed in table 2 and used the Latin hypercube sampling (LHS) method to generate data presented in figure 4. The LHS is a statistical method for generating efficient near-random samples of a multidimensional parameter space. The interval range for each parameter is divided into pieces—1000 in our case—of equal probability, and then exactly one value of the parameter is chosen from each piece. These sampled values for different parameters are randomly matched to generate 1000 samples in the entire parameter space. Figure 4*a*,*c*,*e* show the spread of the values of the corresponding response functions owing to uncertainty in the values of the model parameters. Figure 4*b*,*d*,*f* show the partial rank correlation coefficients (PRCCs) for the model parameters, which demonstrate how sensitive the corresponding response function is to changes in different parameter values.

The optimal vaccination rate *φ*_NE_ (and hence the relative difference between the herd immunity vaccination rate and the Nash equilibrium vaccination rate given by (*φ*_HI_−*φ*_NE_)/*φ*_HI_) depends on the relative cost of vaccination *C*. As an infected individual has about 50% chance of dying from the disease, the cost of infection *C*_i_ is extremely high. It follows that the cost of vaccination relative to the cost of infection *C*=*C*_v_/*C*_i_ is very small. We therefore used a small value *C*=10^−4^ of the relative cost of vaccination for these two response functions.

Not surprisingly, the vaccination rate *φ*, the strength of cultural norms parameter *ξ* and the rate of vaccine wear-off *ω* have the greatest effect on the basic reproduction number $R_{0}$ (figure 4*b*). The negative value of the PRCC for the vaccination rate *φ* means that increasing the value of *φ* results in decreasing the value of the response function (the basic reproduction number $R_{0}$). Conversely, increasing the values of *ξ* and *ω* results in increasing the value of $R_{0}$.

For the game-theoretic model response functions, the most influential parameters are the strength of cultural norms parameter *ξ* and the rate of vaccine wear-off *ω* (the vaccination rate *φ* is no longer a parameter but rather an outcome of the game-theoretic model). While *ω* has a positive PRCC for both game-theoretic response functions, the cultural norms parameter *ξ* has a positive PRCC value for the optimal vaccination rate *φ*_NE_, and a negative PRCC value for the relative difference between the herd immunity vaccination rate and the Nash equilibrium vaccination rate (*φ*_HI_−*φ*_NE_)/*φ*_HI_. This can be explained as follows. As increasing *ξ* results in higher values of $R_{0}$ (cf. (2.5) and figure 4*b*), rational individuals have more incentive to vaccinate given the higher prevalence of the disease. Hence, increasing *ξ* results in higher values of the Nash equilibrium vaccination rate *φ*_NE_. For the very same reason, the resulting optimal vaccination rate is going to be closer to the herd immunity vaccination rate.

The most important implication of the uncertainty and sensitivity analysis we performed is based on figure 4*e*. For sufficiently small values of the relative vaccination cost *C* (we used *C*=10^−4^), the relative difference between the herd immunity vaccination rate and the optimal vaccination rate is almost always less than 1%, and in most cases is less than 0.5% regardless of the values of the epidemiological model parameters. As we mentioned earlier, the relative cost of vaccination is actually very small owing to the extremely high cost of infection *C*_i_. This is a distinct feature of our game-theoretic model of Ebola, which is notably absent from similar models for other infectious diseases. The conclusion is that the optimal vaccination rate is always extremely close to the herd immunity vaccination rate, and this outcome of our game-theoretic model is not affected by the uncertainty in the values of the epidemiological model parameters.

## 4. Discussion

Using the framework of [31], we adopted an epidemiological model of Ebola [3] by incorporating a compartment for vaccinated individuals, and constructed a game-theoretic model of individual-level vaccination decisions. Such a game-theoretic model can be thought of as voluntary participation of individuals in immunization measures. Voluntary vaccination protocols present the following challenge: if a certain proportion of the population is already vaccinated, then the cost (and perceived risk) of vaccination can outweigh the risk of infection. Consequently, achieving herd immunity vaccination levels becomes very difficult with voluntary compliance.

It is not surprising that our findings align with this general theme. We discovered that if the population adopts selfishly optimal vaccination strategies (i.e. the Nash equilibrium vaccination rate *φ*_NE_ in our game-theoretic model), then the population vaccination coverage falls short of the herd immunity levels unless the cost of vaccination is negligible (i.e. zero mathematically). However, what sets the Ebola model apart from other diseases for which this general framework had been applied to is the high fatality rate. The individuals have about 50% chance of dying if contracting Ebola [1,3]. This makes the cost of infection *C*_i_ in the game-theoretic model extremely large, and consequently, the cost of vaccination relative to the cost of infection *C*=*C*_v_/*C*_i_ infinitesimally small. This results in the optimal vaccination rate being almost equal to the herd immunity threshold vaccination rate (figure 3*a*). For example, if *C*=10^−4^, then the optimal vaccination rate is within 1% of the herd immunity vaccination rate (figure 4*e*).

One possible implication of this finding is that a voluntary vaccination programme for Ebola can be extremely effective, and may even result in complete eradication of Ebola if coupled with additional measures such as public education initiatives and monetary incentives or subsidies. Public education initiatives can inform people on the existence, symptoms and spread of Ebola, which would decrease transmissions. Mass media campaigns could potentially encourage individuals who might otherwise not vaccinate to do so [46]. Additionally, collaboration with health officials, spiritual leaders and anthropologists can help replace dangerous ceremonies with safer alternatives [18]. Such programmes would effectively decrease the transmission rate, which studies have shown would be highly effective in reducing the spread of Ebola [17]. Vaccination subsidies or incentives would help drive the (direct) cost of vaccination down, effectively reducing the relative cost of vaccination *C* even further.

The game-theoretic analysis we employed in this paper assumes that individuals are rational and possess perfect knowledge of the population vaccination level and the direct and indirect costs of vaccination and infection. However, this is not the case in reality. This is why it is crucial to use public education measures in order to nudge irrational or misinformed individuals to make rational decisions based on objective information regarding vaccination.

We performed uncertainty and sensitivity analysis of our model using the LHS method (figure 4). The most influential parameters in the epidemiological model were: (i) the vaccination rate *φ*; (ii) the rate of vaccine wear-off *ω*; and (iii) the cultural norms parameter *ξ*. The vaccination rate is not a parameter (but an outcome) of a game-theoretic model, so the rate of vaccine wear-off and the cultural norms parameter had the highest influence on the optimal vaccination rate. The uncertainty analysis of the relative difference between the optimal vaccination rate and the herd immunity vaccination rate as a response function shows that, provided the cost of vaccination relative to the cost of infection is sufficiently small, this relative difference is almost always less than 1%, despite the uncertainty in the epidemiological parameter values.

There are several directions in which our results could be extended. First, the general framework of [31] is based on the static analysis that assumes the system has reached an equilibrium (either a disease-free equilibrium or an endemic equilibrium) before individuals decide on their personal vaccination strategies. However, convergence to the equilibrium state normally occurs on a timescale longer than the one on which individuals make vaccination decisions. The probability of getting infected changes dynamically depending on the disease prevalence. Individuals may have more incentive to vaccinate at the beginning of the epidemic, when the force of infection is high. Modelling such phenomena requires developing dynamic game-theoretic methods where individual vaccination decisions depend on the current state of the system and affect its further evolution. Second, an epidemiological model based on ordinary differential equations assumes a well-mixed population. It does not take into account possibly different interaction (and hence disease transmission) rates between spatially and socially connected groups of individuals. Therefore, it would be beneficial to construct a spatially explicit game-theoretic model of Ebola vaccination decisions (e.g. as a game on a graph) to account for this factor. Moreover, as Ebola cases usually occur as localized outbreaks, employing a dynamic or a spatially explicit model or a combination of both should provide a more realistic view into the effect of individual vaccination decisions on the control and potential eradication of Ebola.

Finally, we made some assumptions on the efficacy of the vaccine based on limited preliminary test data. These assumptions may need to be revisited once the vaccine is released and more actual data are accumulated.

## Data accessibility

This article has no additional data.
